# The Treatment of Sprained Ankle*Remarks at the Atlanta Society of Medicine, November 17, 1896.

**Published:** 1896-12

**Authors:** W. S. Elkin

**Affiliations:** Atlanta, Ga.


### THE TREATMENT OF SPRAINED ANKLE.*

BY W. S. ELKIN, M.D.,
ATLANTA, GA.

I regret that I have not had time to prepare a paper for to-night, as I promised; but I will make a few remarks on this subject anyhow and give you a practical demonstration of the method of treating sprained ankles with adhesive plaster.

We all recognize that a sprained ankle is of frequent occurrence, and realize the fact that the use of plaster-of-paris dressing in the treatment of these injuries is not altogether satisfactory. It is heavy and uncomfortable, often painful, and necessitates the use of crutches, whereas the treatment I am about to detail gives no pain or inconvenience, requires no crutches and is not followed by any loss of function of the joint. The method I advocate was first suggested to me by Dr. V. P. Gibney, of New York, yet he lays no claims to priority, but gives the credit to Mr. Edward Cotterell, of London. All that is needed is a roll of good rubber adhesive plaster, Seabury & Johnson's answers the purpose admirably, a pair of scissors, and a cotton roller bandage two or three yards long and two inches wide. I generally get the three-inch rolls of plaster and cut them or tear them into strips about one inch wide, and from ten to fourteen inches long; the number of strips used will depend upon the size of the foot, and the character of sprain. After the foot has been thoroughly cleansed with soap and water, it should be elevated and vigorously massaged for a few minutes in order to render it more or less bloodless and reduce the swelling; then the strips are applied to the surface of the skin, completely covering the seat of injury. In order to give you a practical demonstration of this method of treatment, I will have this boy remove his shoe and stocking, and put on a dressing for a sprained ankle involving the external malleolus. The same treatment is applicable to the inside of the foot. I begin with my adhesive strip on the outer side of the foot at the base of the little

*Remarks at the Atlanta Society of Medicine, November 17, 1896.

toe, carrying it around the heel and under the plantar arch to the base of the big toe; the next strip begins on the outer side of the leg at the junction of the middle and lower third, is carried down the outer side of the tendo Achilles, under the heel, and up the inner side of the same tendon a few inches above the internal malleolus. Alternate strips are applied, each overlapping the other about one-half its width until the external or injured side of the foot is completely encased. You will observe when the dressing is complete that a small space over the dorsum is not covered with the strip, but is left free intentionally to prevent strangulation should any subsequent swelling occur. If the tarsal or mid-tarsal joints are involved, and the whole foot is swollen, Dr. Gibney suggests that the strips be applied in this manner: Begin the first strip on the inner surface of the heel, carry it back over the heel and over the dorsum of the foot, terminating at the base of the big toe. The second strip starts on the outer side of the heel, below the external malleolus, goes over the heel and dorsum of the foot to the base of the little toe; then other strips are applied in a similar manner until the whole surface is covered in. If the toes be very much swollen, he suggests that they be strapped separately before the other strips are applied. I have seldom found it necessary to adopt this latter method of applying the strips; the other plan meets all indications, and in my hands has proven more comfortable to the patient. After the rubber plaster has been applied, a smooth fitting roller bandage should envelop the foot and ankle, and the patient directed to put on his shoe, walk around the room a few times and then return to his home or business. As soon as the heat of the body causes the plaster to firmly adhere to the surface of the skin, and to adapt itself smoothly to the contour of the foot, the roller bandage can be removed. In the course of a week, or perhaps less time, the strips should be removed and new ones applied, and this continued until the swelling has disappeared and the ankle cured, which generally occurs in a week or ten days. For the past three or four years I have used no other treatment for sprained ankles, both in my hospital and private practice, and have yet to see a case that has not been speedily cured with no subsequent impairment of the function of the joint. Two or three years ago I had the misfortune to sprain my own ankle while trying to board a street-car, and had Dr. Cooper treat me in the manner described, with immediate relief and no loss of time from my work.

Recently a case came under my observation at the Grady Hospital in which the injury was so severe that I hesitated at first to use this treatment. The foot, toes and leg were greatly swollen, with much edema and ecchymosis in the neighborhood of the external malleolus. After satisfying myself that no fracture existed I decided to apply the rubber strips. Although the patient was brought to the hospital in an ambulance, he walked to his place of business after the dressing was applied. I renewed the strips three times in this case, and a perfect cure resulted in three weeks. I am unable to state how this treatment acts, but I am confident you will be pleased with the results obtained from its use.

